# Exploring the Geographic Variation in Fruit and Vegetable Purchasing Behaviour Using Supermarket Transaction Data

**DOI:** 10.3390/nu14010177

**Published:** 2021-12-30

**Authors:** Victoria Jenneson, Graham P. Clarke, Darren C. Greenwood, Becky Shute, Bethan Tempest, Tim Rains, Michelle A. Morris

**Affiliations:** 1 Leeds Institute for Data Analytics, University of Leeds, Leeds LS2 9JT, UK; D.C.Greenwood@leeds.ac.uk (D.C.G.); m.morris@leeds.ac.uk (M.A.M.); 2 School of Geography, University of Leeds, Leeds LS2 9JT, UK; G.P.Clarke@leeds.ac.uk; 3 School of Medicine, University of Leeds, Leeds LS2 9JT, UK; 4 Sainsbury’s Supermarkets Ltd., Holborn, London EC1N 2HT, UK; Becky.Shute@sainsburys.co.uk (B.S.); Bethan.Tempest@sainsburys.co.uk (B.T.); Tim.Rains@sainsburys.co.uk (T.R.)

**Keywords:** supermarket, transaction data, food purchase behaviour, fruit and vegetables, dietary assessment, geographic clustering

## Abstract

The existence of dietary inequalities is well-known. Dietary behaviours are impacted by the food environment and are thus likely to follow a spatial pattern. Using 12 months of transaction records for around 50,000 ‘primary’ supermarket loyalty card holders, this study explores fruit and vegetable purchasing at the neighbourhood level across the city of Leeds, England. Determinants of small-area-level fruit and vegetable purchasing were identified using multiple linear regression. Results show that fruit and vegetable purchasing is spatially clustered. Areas purchasing fewer fruit and vegetable portions typically had younger residents, were less affluent, and spent less per month with the retailer.

## 1. Introduction

Poor dietary quality contributes to rising rates of obesity and associated comorbidities in the UK [1,2]. Many years of policies to encourage individual behaviour change have done little to reverse obesity rates [3]. Moreover, the influence of the food environment on obesity and poor diets [4,5] has attracted policy attention [6]. Measures such as changes to food promotions [7,8] and the soft drinks industry levy [9] in the UK have focused on altering the food environment to ‘nudge’ people towards healthier choices. The food industry has also taken voluntary action to make healthier diets more achievable, such as committing to selling more portions of vegetables as part of the Peas Please campaign [10,11].

Studies of dietary behaviours are important for monitoring population dietary trends and responses to interventions such as policy changes. Population dietary assessment typically employs national survey data, such as the UK’s National Diet and Nutrition Survey (NDNS) [12]. Surveys employ self-report methods, such as food diaries and food frequency questionnaires, and offer detailed information on diet and nutrition as well as participant characteristics. This makes them useful for understanding the socio-demographic determinants of diet [13,14,15,16]. However, the time and cost burdens for participants to complete surveys, and for researchers to code their outputs, limits their sample sizes. Relatively low sample sizes mean that the spatial resolution of national surveys is often poor and rarely offers detail below the regional level; regions in England have an average population greater than five million [17]. This limits their utility to investigate spatial dietary inequalities which often occur at the neighbourhood level.

These surveys enable us to monitor and understand consumption of fruits and vegetables which in turn can be used as a proxy for a healthy diet due to their role in prevention of non-communicable diseases such as cancer [18], due to their richness in beneficial micronutrients, fibre and non-nutritive compounds, and their low energy density. Average fruit and vegetable consumption in the UK is below recommended levels in all ages [19], particularly among low-income groups [19]. Existing dietary inequalities, especially in vegetable intake [20], have further deepened as a result of the COVID-19 pandemic [21], highlighting the need for additional action.

The inequalities in non-communicable disease rates and life expectancy seen at the neighbourhood level [22] suggest that diets may follow spatial patterns similar to those observed for deprivation. This is supported by the food environment literature which considers access to ‘healthy’ and ‘unhealthy’ food outlets. Deprived areas are more likely to display a disproportional density of fast-food outlets [23], and convenience shops, which are less affordable and lack variety in their fruit and vegetable offering [24]. Spatial exploration of supply-side characteristics, such as food environment exposures, is important for revealing inequalities in exposures and have led to planning policies banning fast-food outlets near schools by some local authorities in England [25].

However, previous studies have found the relationship between accessibility to food environment exposures and diet and health outcomes to be non-stationary over space [26,27], suggesting moderation by uncaptured environmental and/or social determinants. As neighbourhood food availability does not necessarily translate to dietary behaviours among the individuals and households who live there, there is a need for large-scale exploration of demand-side diet-related behaviours at the small-area level, which has been lacking previously due to the limited spatial scale afforded by dietary survey data. That said, there is some evidence from survey data that shows that dietary quality varies spatially in line with the socioeconomic gradient. Healthier diets and higher fruit and vegetable intakes were found in neighbourhoods with a higher socioeconomic status in several countries [28,29,30,31]. However, it is not easy to examine diet at the small-area level using traditional dietary assessment approaches, without which much of the local nuance is likely to be missed.

Considering purchases as an upstream behaviour for consumption, supermarket transaction records have been proposed as complementary to dietary surveys [32], with the capacity to provide additional insight as a result of their granularity. Automatically generated electronic food purchase data have the potential to offer large volumes of geocoded information about household food and nutrition availability [33,34,35,36]. Using transaction records for loyalty card holders at a UK supermarket chain, this paper explores small-area and demographic variations in fruit and vegetable purchases (including fresh, frozen and dried varieties) that exist within a single city (Leeds, England). Additionally, we identify determinants of neighbourhood fruit and vegetable purchase levels. Given that areas of similar sociodemographic profile tend to cluster together, we anticipate a spatial patterning of fruit and vegetable purchases. This paper offers a novel exploration of the small-area geography of actual dietary purchase behaviours, as opposed to exposure. Thus, we provide a step towards an incorporated study of both supply and demand, which is likely to provide greater insight into how people’s interactions with their food environments shape their dietary habits. Revealing area-level characteristics which put residents at risk for low purchasing of fruits and vegetables may be used to better understand drivers of diet-related health inequalities and to target local interventions.

This paper will:

1. Examine the small-area spatial distribution of fruit and vegetable purchases and predictors of this purchase behaviour
2. Explore associations at a neighbourhood level between mean daily fruit and vegetable portions purchased and area socioeconomic characteristics, customer demographics, and access to supermarkets.
3. Develop a statistical model that identifies drivers of fruit and vegetable purchasing at a neighbourhood level.

## 2. Materials and Methods

### 2.1. Study Sample

The study sample included 50,917 customers who held a loyalty card for a major UK supermarket, registered to an address in the city of Leeds, England. Eligible customers made at least ten transactions during 2016, which included a minimum of seven out of 16 food categories, developed from categories captured by the Living Costs and Food Survey (LCFS) [37] (Table 1). The inclusion criteria are described in more detail elsewhere [36], but briefly they aim to capture ‘primary’ shoppers who do the majority of their food shopping with the study retailer. The median shopping frequency of our sample is 53 occasions annually (interquartile range 33–82) [36]. Thus, we exclude customers with infrequent purchases from a limited range of food categories, on the basis that their purchases are unlikely to represent their overall diet.

Exploratory data analysis identified some customers with extremely high loyalty card expenditure which we considered unlikely to represent typical household purchasing. We defined an upper bound of annual expenditure, based on household expenditure on food and non-alcoholic beverages from the 2016 edition of the Family Food Survey (FFS) [37]. A threshold of 1.5 times the inter-quartile range beyond the upper quartile (a common criteria to identify large outliers in box plots) from the FFS report, was used to exclude customers at the upper end of the expenditure distribution. For symmetry, the same proportion of customers (1.95%) at the bottom end of the annual expenditure distribution was removed. Customers must be aged 18 or over to obtain a loyalty card with the retailer. For this reason, we excluded customers with a recorded age of 17 years or below as these were assumed to be data errors. Anonymised customer characteristics (age, gender, and output area of residence) were derived from the retailer’s loyalty card sign-up questionnaire. We assume that the loyalty card holder is the main person responsible for shopping in the household.

### 2.2. Study Region

**Figure 1:**
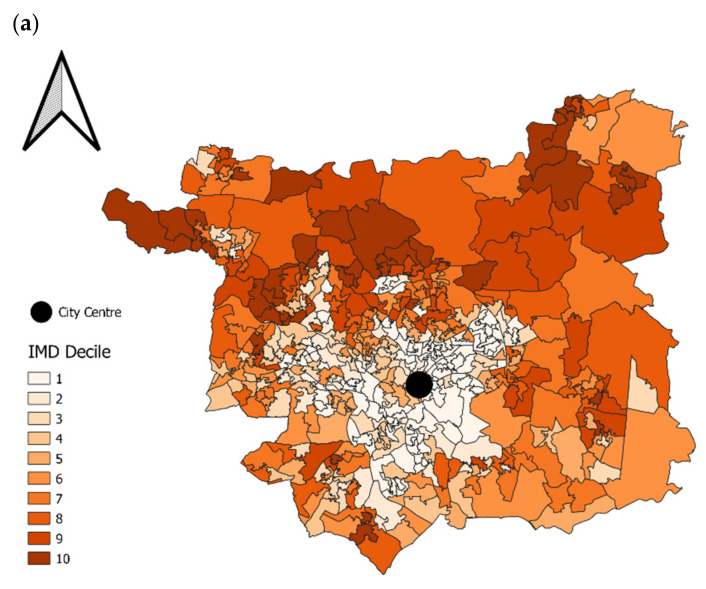

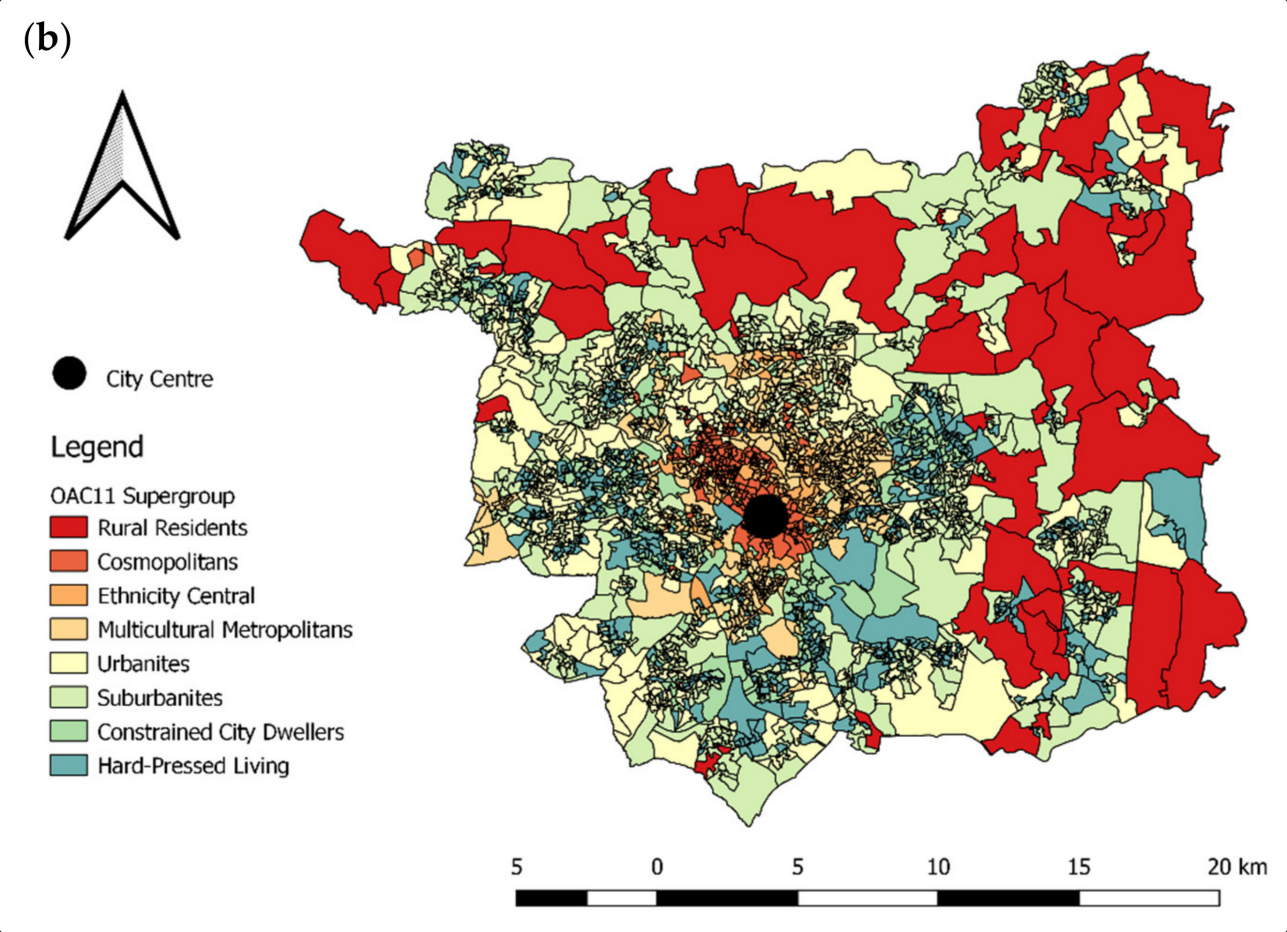
(**a**) Index of Multiple Deprivation decile by Lower Super Output Area in Leeds. (**b**) Output Area Classification by Output Area in Leeds.

The study region is determined by customers whose loyalty card is registered to an output area inside the Leeds boundary. Leeds is a diverse city with cosmopolitan (ethnically diverse) and deprived areas in the south and west of the city, affluent suburbs in the north and east, and a large student population in the inner western suburbs (Figure 1a) shows the spatial distribution of the 2015 Index of Multiple Deprivation (IMD) decile at the Lower Super Output Area (LSOA) level, a neighbourhood census geography representing 400–1200 households. The IMD is a rank of deprivation for more than 32,000 LSOAs in England [38]. These are split into deciles, where 1 represents the most deprived 10% of areas in England. Figure 1b shows the 2011 UK Output Area Classification (OAC) for Output Areas (OAs) in Leeds; the OA is a small-area census geography containing around 125 households. The OAC is an open-source census-derived national hierarchical geodemographic classification system [39,40].

Customer area of residence is known at the Output Area (OA) level and is used to describe the characteristics of areas in the study in the absence of detailed individual-level demographic data. This study uses the Supergroup level of the OAC hierarchy, which assigns areas to one of eight Supergroups, according to the affluence, ethnic composition, rurality, age demographics and other characteristics of the people residing there. Due to small customer numbers at the OA level, areas were aggregated to the Lower Super Output Area (LSOA) (400–1200 households) [41] for analysis. LSOAs with low customer numbers (<*n* = 10) were excluded. The OA of residence centroid was used to assign eastings and northings for customer residential location. This was used to calculate Euclidean (straight-line) distance to the nearest store and most frequently used store, from the study retailer.

### 2.3. Transaction Data

All loyalty card transactions made with the retailer by our ‘primary shopper cohort’ (online and in any store regardless of format, including those made outside of the study region) were collected for the 2016 calendar year. Items in the transaction database have a corresponding weight and number of units, used to calculate their quantity by weight or volume. Non-food and beverage transactions were removed from the database by the retailer prior to access by the research team. Transactions are linked to the loyalty card holder by a unique hashed customer pseudo-ID, and items purchased on a single occasion are linked by a transaction ID.

### 2.4. Estimating Fruit and Vegetable Purchases

Item sub-categories used by the retailer were mapped to categories from the LCFS (Table 1) [37]. Fruit and vegetable purchases were then identified by selecting the relevant LCFS categories (Fruit, and Vegetables and salad) (Table 1). The LCFS is a granular database containing approximately 80 food categories, allowing for the exclusion of potatoes (in line with the UK’s five-a-day fruit and vegetable consumption guidance [42]), and inclusion of both fresh and processed (e.g., frozen and canned) fruits and vegetables. As ready meals were coded as a separate category their constituent parts are not quantified. Therefore, any fruit or vegetables purchased as part of ready meals are not accounted for.

Mean daily fruit and vegetable portions purchased were calculated for each household, by dividing their total purchased weight (grams) in 2016 by 80 (the number of grams recommended as a portion of fresh fruits and vegetables in the UK’s five-a-day recommendation [43]), and then further dividing by 366 (2016 was a leap year). While dried fruits and pulses contributed to the overall fruit and vegetable purchased weight, their recommended portion size (30 g) was not explicitly accounted for, due to challenges in data format allowing accurate identification of them, underestimating their contribution to purchased portions. In the absence of supplementary survey data, food waste and the edible proportions of fruits and vegetables were not accounted for, nor was the number of people living in the household.

### 2.5. Analysis

This study used a multiple linear regression model to identify drivers of mean daily fruit and vegetable purchasing at the neighbourhood level. Model parameters were chosen to represent three domains which were considered to be theoretically influential for dietary choices; customer demographic characteristics (% females, and % aged 65+ years (other age groups were omitted due to lack of influence on the model)); neighbourhood characteristics (mean Index of Multiple Deprivation decile and % of customers in each OAC Supergroup); and accessibility metrics (mean distance to nearest store, mean distance to most-used store, and shopping frequency (mean monthly food and beverage transactions)). Mean total monthly spend on food and beverages (£) was also controlled for.

Outliers from the model were identified as those LSOAs with a Cooks distance (accounting for leverage and residuals) greater than 0.009, using the threshold 4/n (*n* = 439). The model was then reapplied after exclusion of model outliers, which allowed for exploration of the characteristics of outlier neighbourhoods.

Prior to building the regression model, the correlation between mean daily fruit and vegetable portions purchased and each predictor variable was estimated using Kendall’s Tau correlation to inform variable selection for the regression model. Secondly, spatial autocorrelation of each variable was explored using the univariate Moran’s I (Index), to inform the need for a geographically weighted regression model (GWR). LSOAs without any customers were omitted from the Moran’s I calculation. Moran’s I may hold a value from −1 (indicating perfect dispersion) to 1 (indicating perfect clustering), where 0 indicates random dispersion. For the purpose of this study, values smaller than −0.5 are considered as evidence of dispersion, while values greater than 0.5 are considered evidence of clustering, if they are significant at the 95% confidence level. Exploration of the explanatory variables revealed spatial clustering only in those variables which were inherently spatial in nature (IMD and OAC supergroup). For this reason, it was considered that their inclusion in an ordinary least squares (OLS) regression model should be sufficient to capture much of the neighbourhood variation in the outcome, and a GWR model was not used.

## 3. Results

### 3.1. Customer Characteristics

The data cover 50,917 loyalty card holders, equivalent to approximately 6% of the Leeds population. However, as loyalty cards typically represent a household, in reality our sample likely accounts for a larger proportion of residents. Without detailed household size information for the study sample, the exact number of people captured is unknown, but using the average household size for Leeds (2.3 people [44]), we estimate it represents approximately 117,000 people (around 15% of the Leeds population).

A summary of customer characteristics, compared with demographics for Leeds overall, is shown in Table 2. The number of female loyalty card holders was more than double the number of male loyalty card holders. Almost 40% of customers are in the 45–64 age band, which is over-represented compared with the Leeds population. The sample over-indexes on customers living in affluent regions; more than 72% of customers live in LSOAs in the five most affluent deciles, compared with less than 43% of the general Leeds population. Compared with the population of Leeds, the customer sample over-indexes on customers from Rural Residents, Urbanites and Suburbanites, and under-represents people from areas classified as Cosmopolitans, Ethnicity Central, Multicultural Metropolitans, Constrained City Dwellers, and Hard-pressed Living supergroups.

### 3.2. Fruit and Vegetable Purchases in Leeds

In 2016, customers across Leeds purchased on average 3.4 portions (equivalent to 272 g) of fruit and vegetables per household per day (Table 2). This is equivalent to around 1.5 portions per person per day, given the average household size of 2.3 persons [44]. Mean fruit and vegetable portions when aggregated across LSOAs was lower at 3.0/household/day (Table 3), highlighting that accounting for local averages can mask local patterns. Female loyalty card holders purchased on average 0.23 portions more per day for their household than males. Younger adults purchased fewer daily portions per household of fruits and vegetables (mean = 2.96 per for 18–44 years) compared with older adults (mean = 3.64 for adults age 65+). Customers living in the most deprived areas (IMD decile 1, mean = 2.80 portions per household) purchased on average 1.12 portions per household of fruits and vegetables fewer each day compared with customers in the most affluent areas (IMD decile 10, mean = 3.92 portions per household). Customers living in Suburbanite areas had the highest purchases of fruits and vegetables (3.77 portions/household/day), while those in Cosmopolitan areas purchased the fewest portions (2.58 portions/household/day), a difference of 1.19 daily portions.

### 3.3. Neighbourhood Characteristics

The characteristics of study areas (aggregated to the LSOA level) are summarised in Table 3. On average, customers in each LSOA live a median of 1.7 km from their nearest study retailer store (which may be a superstore or convenience format) but shop most often at stores further away (a median of 11.2 km away). The average spend with the retailer across LSOAs is £104 per month. Customers have a median shopping frequency with the retailer of just over 5 occasions each month, indicating relative loyalty to the study retailer.

The outcome variable, mean daily fruit and vegetable portions, shows evidence of spatial clustering (Moran’s I 0.52, *p* < 0.001). Evidence of spatial clustering was also found for IMD decile (Moran’s I 0.61, *p* < 0.001), % customers in the Cosmopolitan, Ethnicity Central and Multicultural Metropolitans supergroups (Moran’s I = 0.71, 0.58 and 0.60 respectively, all *p* < 0.001), and mean distance to nearest store (Moran’s I 0.83, *p* < 0.001), while mean total monthly spend was borderline (Moran’s I 0.49, *p* < 0.001). As no evidence of spatial clustering or dispersion was found for the other predictor variables, we accept the null hypothesis that their spatial distribution is random.

Mean total monthly spend (£) was found to be significantly correlated with the outcome (mean daily fruit and vegetable portions/household), indicating that as monthly expenditure increases so does the number of fruit and vegetable portions purchased (C = 0.7, *p* < 0.001). The correlation between IMD decile and mean daily fruit and vegetable portions was also positive and reached statistical significance at the 95% level, though moderate in strength (C = 0.5, *p* < 0.001), indicating that as affluence increases the number of fruit and vegetable portions purchased increases.

### 3.4. Spatial Patterns in Fruit and Vegetable Purchasing

**Figure 2:**
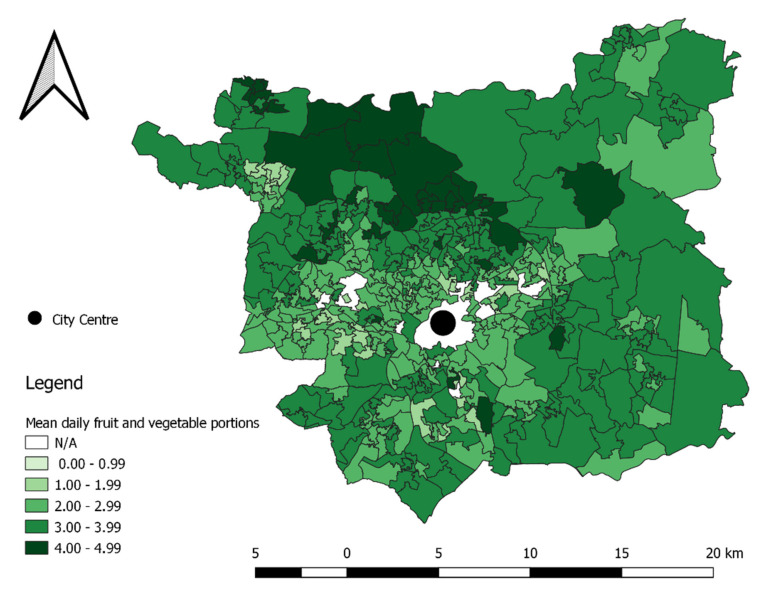
Fruit and vegetable purchasing in Leeds by Lower Super Output Area: mean daily portion per household reas with *N* < 10 customers omitted from map (shown as N/A in the figure legend).

**Figure 3:**
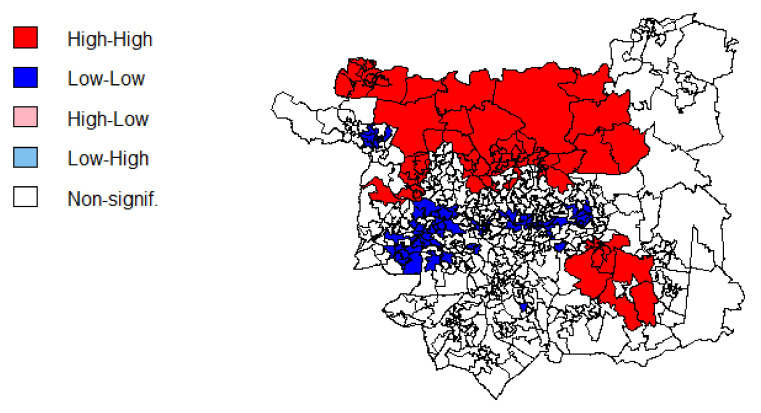
Local Moran’s I for daily fruit and vegetable portions per household.

Figure 2 shows the spatial pattern of fruit and vegetable purchases across Leeds at the LSOA-level. Fruit and vegetable purchasing is spatially clustered (Figure 3) and follows the expected deprivation trend, with the most deprived purchasing fewer fruit and vegetable portions. Households living in the north of Leeds purchase on average four or more portions/day of fruit and vegetables. The more multicultural and urban areas in the centre and south-west of Leeds purchase the fewest daily fruit and vegetable portions. Those more rural and suburban areas surrounding the city centre, particularly to the north and east, purchase 3–4 portions/day on average per household.

### 3.5. Linear Regression

Regression coefficients for all LSOAs (*n* = 439) are shown in Table 4. Lower deprivation and a greater proportion of older adults (65+ years) are positively associated with mean daily fruit and vegetable portions per household purchased at the LSOA level. Mean daily fruit and vegetable purchases among LSOAs in IMD decile 10 (the least deprived) are around 0.45 portions per household higher than the most deprived LSOAs (IMD decile 1). Theoretically, an area where 100% of the population are aged 65 years or older is likely to purchase half a portion/household/day more fruits and vegetables on average than an area where only 1% of the population are aged 65+. The proportion of female customers did not affect household fruit and vegetable purchasing at the LSOA-level, but it was influenced by output area classification. A higher proportion of customers living in neighbourhoods classified as Cosmopolitans, Ethnicity Central, Multicultural Metropolitan and Suburbanites, was significantly associated with higher fruit and vegetable purchases, while Constrained City Dwellers were associated with fewer fruit and vegetable portions. A higher mean total monthly expenditure with the retailer was associated with a greater number of fruit and vegetable portions purchased. A £1 increase in LSOA-level mean total food and non-alcoholic beverage spend with the retailer was associated with an additional 0.03 portions fruits and vegetables/household/day purchased. Shopping frequency and distance to store were not associated with fruit and vegetable purchase levels.

Twenty five outlier LSOAs were identified by the model and are summarised in Supplementary Table S1 and mapped in Supplementary Figure S1. Overall, outlier areas had a higher proportion of customers in the most deprived IMD decile, and decile 4, and a lower proportion of customers in the least deprived deciles, compared with the overall sample. These areas included deprived areas with higher fruit and vegetable purchases than expected and low deprivation areas with lower fruit and vegetable purchases than expected. Examination of the group and supergroup levels of the OAC classification also revealed that outlier areas were also more likely to be resided by ethnic minority communities.

**Figure 4:**
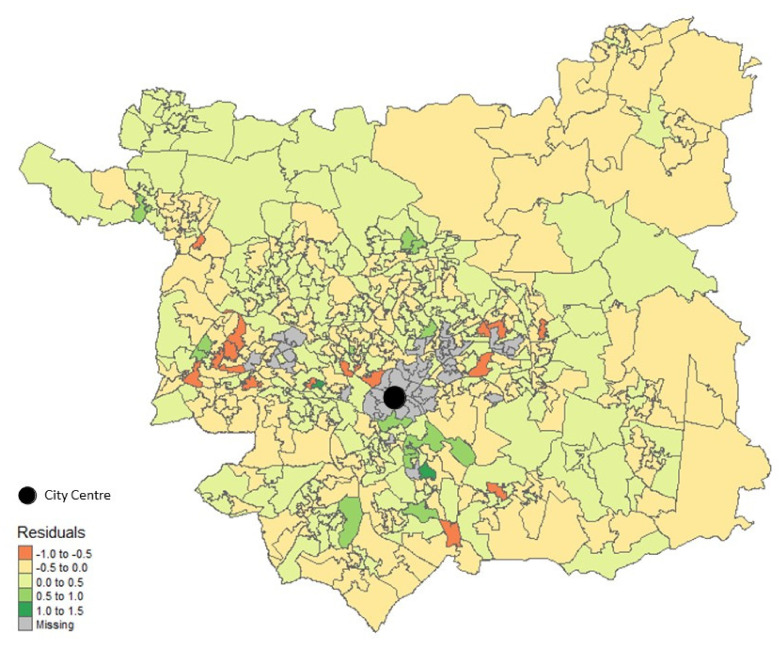
Map of residuals from Model 1.

LSOAs with high positive residual values (Figure 4) (≥0.5), indicating that customers in these areas purchase upwards of 0.5 portions more than predicted, tended to be dominated by OAC sub-groups characterised by families and ethnic minority groups. While those with high negative residuals (≤−0.5), indicating they purchase at least 0.5 portions fewer than predicted, tended to be dominated by OAC sub-groups characterised by retirement living or students, or families with a below average spend.

## 4. Discussion

To the best of our knowledge, this is the first study to examine neighbourhood spatial variation in food purchases using electronic supermarket transaction records. Additionally, the ability to explore diet-related behaviours at such a fine geographic scale is a novel characteristic of purchase records. This study has several strengths including the large sample size which affords statistical confidence in the results; geocoded dietary purchase data permitting visualisation and data linkage at the small-area level; objective dietary purchase estimates free from subject reporting biases; and longitudinal dietary purchase data for a whole year representing habitual dietary behaviours. Our findings demonstrate how novel exploration of large-scale purchase records at the neighbourhood geography level can offer an economical approach to population-level dietary assessment. Detecting socio-spatial influencers of dietary behaviours contributes to knowledge of localised dietary inequalities which are important for identifying potential intervention target areas.

Demographic information is available for this study thanks to loyalty card information provided by the retailer and linkage with area-level demographic data. This enables assessment of sample representativeness, which is noted as important [48] and lacking [49] in previous applications of transaction data for public health nutrition research. The customer samples are mostly female, with an older age distribution than Leeds as a whole. Affluent urban and suburban communities are over-represented while ethnically diverse communities are under-represented. Loyalty card customers introduce sampling bias, yet as a major cohort of the customer base, they make a useful research population. Despite the myth, surveys are not always more representative and tend to under-represent hard-to-reach low-income groups, especially those that use random sampling [50,51]. While some small geographic areas in this study have low customer numbers, the overall sample (n > 50,000) is very large compared with many presented in the literature and all socio-economic and geodemographic groups are represented in relatively large numbers (the lowest being 731 customers in the Ethnicity Central Output Area Classification Supergroup). Supermarket data, even from a single retailer, may therefore contain higher numbers of the hard-to-reach groups, giving greater power across all socioeconomic segments of the population. That said, we cannot be sure that customers in our sample are typical of their neighbourhood characteristics.

Customers in Leeds purchased on average 3.4 portions of fruits and vegetables per household per day, which equates to just 1.5 daily fruit and vegetable portions per person, considering the size of the average Leeds household (2.3 people) [44]. Our purchase estimate is well below the five-a-day recommendation and lower than daily intakes estimated by the NDNS (4.2 portions per person) [52] and the Health Survey for England (HSE) (3.8 portions per person) [53]. Survey estimates are known for over-reporting of fruit and vegetables due to social desirability biases, which are not a problem for objective automated purchase records.

The degree to which household-level purchases from the retailer represent individual consumption is unknown. Previous validation studies highlight that agreement between purchases and consumption is likely to vary by loyalty status and household composition [54,55], with higher agreement observed for single-person households [54]. However, accepted adjustment factors remain lacking. Future work could incorporate known dietary variation by gender and life-stage by accounting for household composition (number and age of household members) to more accurately estimate individual-level intake from household purchase records. As this information cannot typically be obtained from retailer loyalty card records, this may involve using survey data, area-level estimates, or the development of methodologies to model household composition, for example microsimulation using census statistics [56,57].

As we do not account for household waste or inedible proportions, our portions estimate may be inflated by as much as 28% for fresh vegetables and salad, and 6% for fresh fruit, according to national household waste estimates [58]. While robust methods for adjusting transaction records for waste are needed, crude application of national estimates would reduce our portions estimate to roughly 1.1 portions purchased per person per day. Furthermore, as our estimate is from a single retailer only, and does not include fruit and vegetables purchased or obtained elsewhere (e.g., from other retailers, home-grown, or consumed in restaurants) or in composite dishes purchased from the retailer, it is likely to under-represent total household fruit and vegetable purchases.

Fruit and vegetable purchases were found to vary spatially, with clusters of high fruit and vegetable purchasing in the affluent rural and suburban areas to the north and east of the city, while clusters of low fruit and vegetable purchasing were observed in the more deprived neighbourhoods in and around the city centre. The observed association between fruit and vegetable purchasing and area deprivation concurs with research into the geography of dietary patterns based on survey data, which found a higher prevalence of the vegetable-rich ‘health conscious’ and ‘high diversity vegetarian’ dietary patterns in suburban areas with lower deprivation [31,59]. Using transaction records, fruit and vegetable purchases were important determinants of the observed ‘Fruity’ and ‘Meat Alternative’ dietary patterns, which were more prevalent among customers in the most affluent deciles [36]. Yet, it is possible that the observed deprivation pattern may be confounded by differences in household composition, for example the mix of adults and children.

Despite the apparent presence of an overall deprivation gradient in fruit and vegetable choice behaviours, exploration of LOSAs classed as outliers and with high residual values identified neighbourhoods which appear to be exceptions to the rule. These areas suggest that education and ethnicity moderate the effect of deprivation. In spite of relative deprivation and a low overall spend, outlier areas occupied by students and minority ethnic families spent a higher-than-average proportion of their total expenditure on fruits and vegetables, which translated to more portions purchased than predicted. This could be indicative of a preference for scratch-cooking or meal assembly (e.g., the addition of peppers to a fajita meal kit) among these groups. Similarly, deprivation did not translate to low fruit and vegetable purchases for some rural communities. A higher than average spend observed in these outlier areas could be attributed to transactions capturing a larger proportion of total purchases, due to less retail competition. Despite spending a lower proportion of their total expenditure on fruits and vegetables, this did not translate to fewer portions, which may indicate thriftiness and a preference for cheaper fruit and vegetable varieties, which enable them to get more portions for their money.

Outlier LSOAs with lower than predicted fruit and vegetable purchases were occupied by families right across the deprivation spectrum. While these areas had a higher than average spend with the retailer, they prioritised spend on fruits and vegetables to a lesser degree. This may be indicative of busy family lives and a preference for convenience meals, a tendency to source fruits and vegetables elsewhere e.g., greengrocers or home-growing, or a preference for more expensive varieties. Outlier LSOAs also had a lower proportion of female customers overall, especially among more deprived areas. A sensitivity analysis repeating the model after exclusion of outlier LSOAs led to the proportion of females becoming a significant negative predictor of fruit and vegetable purchases (Supplementary Table S2). This is surprising given that females purchase more fruit and vegetables than males on average at the customer-level. While the reason is unclear, it could be that females are more likely to be the primary shopper for busy families which rely on convenience meals.

At the neighbourhood level, a higher proportion of over 65s was associated with higher fruit and vegetable portions purchased. The relationship with age may be a true reflection of differences in fruit and vegetable intake and agrees with other studies which found higher fruit and vegetable consumption among older adults [19,60,61]. Yet, at the household level it is perhaps counter-intuitive that older adults should purchase more portions of fruit and vegetables, given that they are more likely to live alone or with just one other as children have left home. It is possible therefore that the relationship may also reflect differences in purchasing and food preparation practices. For example, younger adults often lack cooking skills, are likely to be under greater time-pressures due to work and childcare responsibilities, and may therefore prefer to choose convenience meals rather than cooking from scratch [62,63]. While estimates by the retailer indicate that ready meals contribute only a small fraction of all vegetables purchased (unpublished data), our inability to accurately quantify the fruit and vegetable content of composite foods is likely to under-estimate fruit and vegetable purchases particularly among low-income working families and young people. Younger adults also consume more takeaway and restaurant meals [13], which may provide additional uncaptured fruit and vegetable portions.

Some research suggests that greater access to supermarkets is associated with higher fruit and vegetable intake [27,28]. Despite this, distance to nearest store and most used store were not found to be significantly associated with fruit and vegetable purchases in either model in this study. Indeed, rural and suburban areas to the north of the city demonstrated both the greatest average distances to nearest store and the highest fruit and vegetable purchases. It is possible that the relationship between proximity and fruit and vegetable purchases may vary spatially, moderated by unmeasured structural factors such as car ownership, access to public transport, store format (superstore or convenience store), the availability of other food outlets in the neighbourhood, and the degree to which a particular retailer meets a customer’s social, cultural and economic needs [27]. While all store formats offer some fruits and vegetables, there will be differences in the range offered. Aggarwal et al. [60] found that only one third of participants shopped at their nearest store, and those who shopped at low-cost stores were more likely to travel beyond their nearest store.

In another study by Liese et al. [64], access to store was associated with frequency of shopping trips, but not with fruit and vegetable intake, suggesting that access may be more closely associated with purchase pattern (e.g., top up shopping compared with a large weekly shop) than purchased amounts. While shopping frequency was not found to be significantly associated with fruit and vegetable purchases in the present study, we observed a narrowing of confidence intervals around our estimates after removal of outlier LSOAs, increasing the significance of findings (supported by a smaller *p*-value). Outlier areas were on average further from their most used store than the sample as a whole. The validity of distance as a measure of access should also be considered as it disregards the store offering and product prices. The average distance to the most-used store was high in this study (>10 km), with a number of customers frequenting stores outside of the Leeds study region. While these are likely to be edge cases led by store network accessibility, this behaviour warrants further exploration. The high distance to the most-used store may be explained, for example, by customers shopping on their commute to work outside of the area, spending time at two addresses (for example students who return home outside of term time), or customers who have migrated outside the area without updating the address associated with their loyalty card.

The literature indicates good agreement between supermarket purchase data and self-reported dietary measures [55,65,66]. Among loyal customers, even a single retailer can make a significant contribution to total household food purchases [33,55,67]. While we do not know how much of a customer’s total purchases are represented by the retailer, we have tried to select a relatively loyal customer sample, as indicated by their membership in the loyalty card scheme and frequent and broad-ranging purchase history. Customers in the sample visit the store on average five times per month. Controlling for total monthly spend on food and non-alcoholic beverages with the retailer goes some way to account for loyalty, assuming that higher spend with the retailer represents a higher proportion of the available food purse. However, higher total monthly spend may also be indicative of a larger household size or affluence, denoting a preference for more expensive premium food stuffs rather than volume of food purchased. Degree of loyalty could better be controlled for using estimates of basket share or the Recency, Frequency, and Monetary value (RFM) index for example. Alternatively, as proposed by Rains and Longley [48], purchase ‘completeness’ at the category level could be estimated by comparing retail expenditure with estimates in national survey data.

While we observed spatial clustering of the outcome variable, the only predictor variables which showed spatial clustering were IMD and OAC, which are inherently spatial. As the deprivation index and geodemographic segmentation to go some way to capturing the nature of the food environment and the characteristics of people who live in an area, we considered the effect of uncaptured spatial factors on the model coefficients to be minimal. Despite this, we found LSOAs with high positive residual values to be clustered in the south of the city and those with high negative residual values to be clustered in the west. Similarly, Clary et al. [27] found nonstationarity in the interaction between food environmental exposures and fruit and vegetable intake using GWR across four London boroughs. While there are likely to be limits to the validity of GWR at such granular geographic scales as that applied in this study, it is possible that our global model may have missed spatial variation in the local food environments and the way in which people respond to their environment. Incomplete spatial representation of dietary behaviours due to missing information about transactions from other retailers further limits the applicability of GWR approaches. Nevertheless, exploration of outlier areas from the regression model revealed some interesting insights which became more apparent when applying more granular levels of the hierarchical Output Area Classification (Group and Sub-group, rather than Supergroup as used in the model).

### 4.1. Policy Relevance

Dietary research has long shown socioeconomic inequalities. While low overall fruit and vegetable purchase level warrant efforts to increase purchasing across the board, geographically untargeted strategies require huge investment and are likely to widen inequalities. To ensure those who purchase the least fruits and vegetables are not left behind, it is important to understand where best to focus interventions. Exploring neighbourhood-level fruit and vegetable purchases offers retailers insights for store-level stocking and marketing decisions. Interventions to increase fruit and vegetable purchases should target stores in areas with low purchase levels, especially those serving younger more deprived urban communities. These areas tend to be served by smaller stores where limited ranges make groceries comparatively more expensive. With small stores set to be exempt from new location-based in-store promotional restrictions in the UK [7], strategies to level the playing field are increasingly important. Strategies focusing on convenience, affordability and appeal are most likely to be successful among these groups [68].

Outliers in the study reveal that the influence of deprivation may be moderated by education and ethnicity, while busy family lives could be an important barrier to purchasing fruit and vegetables. Outlier areas should be explored in more detail in subsequent studies to understand the local factors which cause them to buck the deprivation trend. This evidence would inform the current social prescribing debate by revealing local influencers of healthy diets. Further work should also explore whether diet-related inequalities are contributing to the spatial inequalities which can be observed in a wide range of health outcomes.

### 4.2. Future Directions

Exploration of population diet using electronically captured secondary purchase data is in its relative infancy and, as such, we acknowledge several limitations which set out a foundation for future research. Future directions include estimation of and controlling for household characteristics to extrapolate individual-level estimates; controlling for the inedible proportion of fruit and vegetables and food waste; estimating the fruit and vegetable content of composite dishes; exploring purchases of fruits and vegetables separately, breaking these down further by type; and exploring the effect of seasonality on purchasing behaviours. The validity of applying geographically weighted regression to neighbourhood level geographies, and the ability of existing survey data to completement supermarket purchase records for the development of small area estimation models, should also be considered.

## 5. Conclusions

In conclusion, supermarket loyalty card transactions allow us to investigate small area patterns in food purchase behaviours and reveal that areas purchasing fewer fruit and vegetable portions typically had younger residents, were less affluent, were closer to the supermarket but shopped less frequently, and had a lower total monthly spend with the retailer. In addition, we were able to unpack outliers such as those populated by students which had higher than expected fruit and vegetable purchases despite relative deprivation, illustrating that more nuanced relationships exist than those reported in earlier research.

## Figures and Tables

**Table 1:** Food categories in the transaction database used for sampling.

Category Description ^1^	LCFS Category (LCFS Code)
Carbohydrate products	Bread and cereals (1.1.1)
Cakes and biscuits	Buns, cakes, biscuits etc (1.1.3)
Meat and fish	Meat (1.1.5–1.1.10), Fish (1.1.11)
Dairy	Milk, cheese, eggs (1.1.12–1.1.15)
Fats	Oils and fats (1.1.16–1.1.18)
Fruit	Fruit (1.1.19–1.1.22)
Vegetables and salad	Vegetables (1.1.23–1.1.27)
Potato	Potatoes (1.1.26)
Sweets	Sugar, jam, honey, chocolate confectionary (1.1.28–1.1.32)
Other (e.g., spices)	Other foods (1.1.33)
Non-alcoholic beverages	Non-alcoholic beverages (1.2)
Alcoholic beverages	Alcoholic beverages (2.1)
Ready foods	N/A—additional category not present in the LCFS
Baby food	N/A—additional category not present in the LCFS
Crisps and nuts	N/A—additional category not present in the LCFS
Meat free and free from foods	N/A—additional category not present in the LCFS

^1^ Categories based on the Living Costs and Food Survey categories.

**Table 2:** Coverage of study sample by demographic group, in relation to Leeds and UK.

		Number (%)		
Characteristic		Study Population	Leeds Population ^1^	Mean Daily Portions of FV Purchased Per Household (SD)
Whole sample		50,917 (100.0)	751,485 (100)	3.40 (3.06)
Gender	Male	14,539 (28.6)	367,933 (49.0)	3.22 (2.98)
	Female	32,342 (63.5)	383,550 (51.0)	3.45 (3.10)
	Unknown	4036 (7.9)	-	3.69 (3.07)
Age band	18–44	16,268 (32.0)	269,582 (35.9)	2.96 (2.80)
	45–64	19,614 (38.5)	172,964 (23.0)	3.58 (3.27)
	65+	10,817 (21.2)	109,598 (14.6)	3.64(2.99)
	Unknown	4218 (8.3)	-	3.65 (3.04)
IMD decile	1	3621 (7.1)	186,995 (23.8)	2.80 (2.57)
	2	2035 (4.0)	75,224 (9.6)	2.70 (2.47)
	3	2669 (5.2)	70,571 (9.0)	2.77 (2.56)
	4	1903 (3.7)	33,388 (4.3)	2.86 (2.81)
	5	3769 (7.4)	83,694 (10.7)	2.92 (2.66)
	6	4770 (9.4)	68,864 (8.8)	3.20 (3.00)
	7	7650 (15.0)	89,670 (11.4)	3.48 (3.11)
	8	7573 (14.9)	63,366 (8.1)	3.47 (3.10)
	9	8974 (17.6)	62,882 (8.0)	3.84 (3.26)
	10	7953 (15.6)	50,192 (6.4)	3.92 (3.33)
Output area Classification Supergroup	Rural Residents	1428 (2.8)	12,844 (1.6)	3.58 (3.11)
	Cosmopolitans	3839 (7.5)	80,788 (10.3)	2.58 (2.39)
	Ethnicity Central	731 (1.4)	28,615 (3.7)	2.88 (2.48)
	Multicultural Metropolitans	4889 (9.6)	140,250 (18.0)	3.21 (2.98)
	Urbanites	14,784 (29.0)	161,993 (20.7)	3.50 (3.10)
	Suburbanites	18,445 (36.2)	160,366 (20.5)	3.77 (3.27)
	Constrained City Dwellers	1949 (3.8)	71,244 (9.1)	2.67 (2.47)
	Hard-pressed Living	4852 (9.5)	124,987 (16.0)	2.88 (2.72)

^1^ Leeds population figures (gender and age) from the 2011 UK census, *n* = 751,485 residents [45]. IMD data from 2015/16 by LSOA, *n* = 784,846 residents [46]. OAC Supergroup population estimates derived from 2016 mid-year population estimates (*n* = 781,087 residents) [47]. FV = Fruits and Vegetables.

**Table 3:** Overview of variables at Lower Super Output Area level.

Characteristic of Loyalty Card Holder	Mean (SD)^1^ Median (IQR)	Univariate Moran’s I (Clustering)	*p*-Value (Moran’s I)	Kendall’s Tau rank Correlation with Outcome	*p*-Value (Kendall’s Tau)
Outcome variable					
Mean household daily portions of FV purchased	3.0 (0.7)	0.5	0.001	-	-
Predictor variable					
female (% of sample)	63.6 (8.1)	0.1	0.006	0.0	0.515
aged 18–44 years (% of sample)	34.3 (15.3)	0.4	0.001	−0.3	<0.001
% aged 45–64 years (% of sample)	38.6 (9.8)	0.2	0.001	0.1	0.002
% aged 65+ years (% of sample)	19.1 (9.8)	0.3	0.001	0.3	<0.001
IMD decile	5.2 (3.1)	0.6	0.001	0.5	<0.001
Rural Residents (% of sample)	0.0 (0.0, 0.0)^1^	0.3	0.001	0.2	<0.001
Cosmopolitans (% of sample)	0.0 (0.0, 0.0)^1^	0.7	0.001	−0.1	0.066
Ethnicity Central (% of sample)	0.0 (0.0, 0.0) ^1^	0.6	0.001	−0.1	<0.001
Multicultural Metropolitans (% of sample)	0.0 (0.0, 20.3) ^1^	0.6	0.001	−0.1	<0.001
Urbanites (% of sample)	0.0 (0.0, 41.6) ^1^	0.3	0.001	0.2	<0.001
Suburbanites (% of sample)	0.0 (0.0, 45.3) ^1^	0.4	0.001	0.4	<0.001
Constrained City Dwellers (% of sample)	0.0 (0.0, 7.1) ^1^	0.2	0.001	−0.3	<0.001
Hard-pressed Living (% of sample)	0.0 (0.0, 24.4) ^1^	0.2	0.001	−0.2	<0.001
Mean distance to nearest store (km)	1.7 (0.9, 2.8) ^1^	0.8	0.001	0.1	<0.001
Mean distance to most used store (km)	11.2 (6.4, 17.6) ^1^	0.4	0.001	−0.1	<0.001
Mean total monthly spend (£)	104.3 (19.1)	0.5	0.001	0.7	<0.001
Shopping frequency (mean monthly trips)	5.0 (4.4, 6.0) ^1^	0.4	0.001	−0.1	<0.001

^1^ For variables which did not display a normal distribution, the median and interquartile range (IQR) are the provided summary statistics. FV = Fruits and Vegetables.

**Table 4:** Results of OLS ^1^ regression predicting household fruit and vegetable purchasing (portions/day).

	OLS Regression, *n* = 439 LSOAs (Adj R2: 85.8%)	
Variable ^2^	Coefficient (95% CI)	*p*-Value
Intercept	−0.565 (−0.918, −0.213)	0.003
Mean monthly spend (£)	0.031 (0.029, 0.032)	<0.001
% aged 65+ years	0.005 (0.002, 0.008)	0.002
IMD decile	0.045 (0.028, 0.061)	<0.001
Shopping frequency (mean monthly trips)	0.026 (−0.001, 0.053)	0.066
% female	−0.003 (−0.007, −0.000)	0.057
Distance to nearest store (km)	0.006 (−0.021, 0.033)	0.654
Distance to most-used store (km)	0.001 (−0.001, 0.003)	0.280
% Rural Residents	−0.003 (−0.006, 0.001)	0.126
% Cosmopolitans	0.003 (0.001, 0.005)	0.011
% Ethnicity Central	0.004 (0.001, 0.007)	0.005
% Multicultural Metropolitans	0.002 (0.001, 0.003)	0.003
% Urbanites	0.002 (0.001, 0.003)	0.008
% Suburbanites	0.001 (−0.001, 0.002)	0.436
% Constrained City Dwellers	−0.002 (−0.003, 0.000)	0.093

^1^ OLS = Ordinary Least Squares. ^2^ % OAC 8 (Hard-pressed living) was excluded from the model due to perfect multicollinearity with the intercept. IMD: Index of Multiple Deprivation.

## Data Availability

Due to the commercial nature of the data used in this research, it is not possible for data to be published alongside the manuscript.

## Supplementary Materials

The following are available online at https://www.mdpi.com/article/10.3390/nu14010177/s1, Table S1: Outlier LSOAs (*n* = 25) by IMD decile; Table S2. Sensitivity analysis showing results of regression model after exclusion of outlier LSOAs; Figure S1: Map of Outlier LSOAs from Model 1 (*n* = 25).
