# Supplementary material for: Exploring the Geographic Variation in Fruit and Vegetable Purchasing Behaviour Using Supermarket Transaction Data
Source: Nutrients. 2021 Dec 30;14(1):177. doi: 10.3390/nu14010177 (PMC8747042; doi:10.3390/nu14010177)
Supplement: Supplementary file 1 [file nutrients-14-00177-s001.zip › nutrients-1505122-supplementary.pdf]

Supplementary Table S1. Outlier LSOAs (n=25) by IMD decile.

IMD decile	Mean daily FV portions	% outlier customers
1	2.88	40.00%
2	2.82	8.00%
3	2.69	8.00%
4	2.97	12.00%
5	2.75	8.00%
6	3.27	8.00%
8	3.49	8.00%
9	2.77	8.00%
10	N/A	0.00%
TOTAL	2.93	100.00%

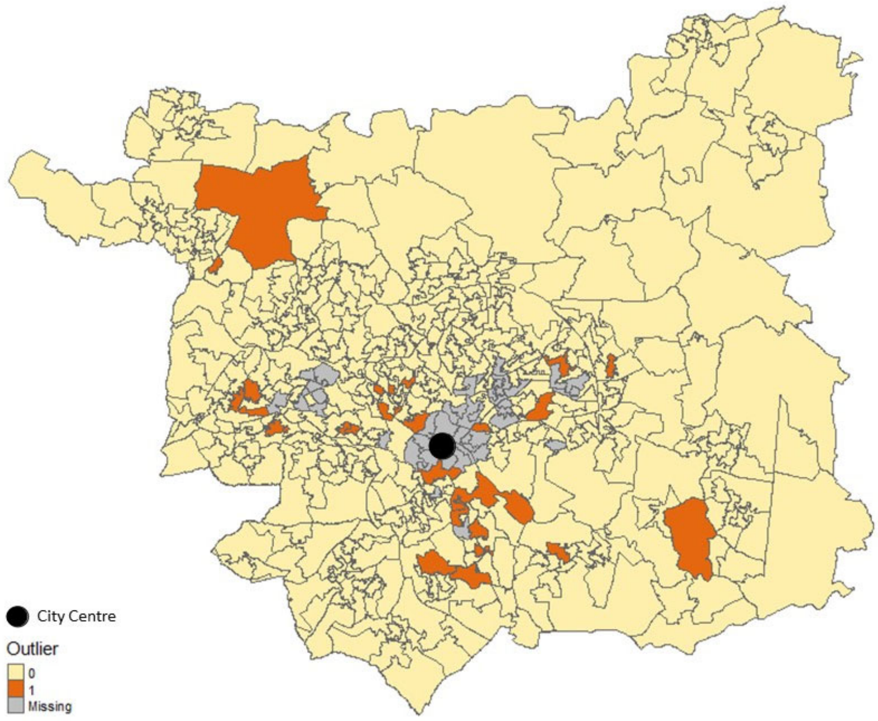

Supplementary Figure S1. Map of Outlier LSOAs from Model 1 (n=25)[1].

[1]1 indicates outlier areas according to Cooks Distance threshold 0.009

Supplementary Table S2. Sensitivity analysis showing results of regression model after exclusion of outlier LSOAs.

	**OLS regression, Model 2 n=414 LSOAs (Adj R2: 90.4%)**	
**Variable[1]**	**Coefficient (95% CI)**	**P-value**
Intercept	-0.434 (-0.732, -0.136)	0.006
Mean monthly spend (£)	0.031 (0.030, 0.033)	<0.001
% aged 65+ years	0.006 (0.003, 0.008)	<0.001
IMD decile	0.053 (0.039, 0.067)	<0.001
Shopping frequency (mean monthly trips)	0.007 (-0.016, 0.029)	0.577
% female	-0.005 (-0.008, -0.002)	0.003
Distance to nearest store (km)	-0.000 (-0.023, 0.023)	0.982
Distance to most-used store (km)	0.001 (-0.001, 0.002)	0.554
% Rural Residents	0.006 (0.003, 0.009)	<0.001
% Cosmopolitans	0.003 (0.001, 0.005)	0.003
% Ethnicity Central	0.003 (0.000, 0.006)	0.028
% Multicultural Metropolitans	0.002 (0.001, 0.003)	0.004
% Urbanites	0.001 (-0.000, 0.002)	0.094
% Suburbanites	-0.001 (-0.002, 0.001)	0.419
% Constrained City Dwellers	-0.002 (-0.003, 0.000)	0.075
